# *Epimedium* Extract Promotes Peripheral Nerve Regeneration in Rats

**DOI:** 10.1155/2013/954798

**Published:** 2013-09-17

**Authors:** Yuhui Kou, Zhiyong Wang, Zhihong Wu, Peixun Zhang, Yu Zhang, Xiaofeng Yin, Xisheng Wong, Guixing Qiu, Baoguo Jiang

**Affiliations:** ^1^Department of Orthopedics, Peking Union Medical College Hospital, Chinese Academy of Medical Sciences and Peking Union Medical College, Beijing 100730, China; ^2^Department of Trauma Orthopedics, People's Hospital of Peking University, Beijing 100044, China

## Abstract

Effects of *Epimedium* extract and its constituent icariin on peripheral nerve repair were investigated in a crush injury rat model. Animals were divided into four groups: sham, control, *Epimedium* extract, and icariin groups. At postoperative weeks 1, 2, 4, and 8, nerve regeneration and functional recovery were evaluated by sciatic functional index (SFI), nerve electrophysiology, nerve pinch test, and muscle wet weight. Results showed that at 2 and 4 weeks after surgery rats in the *Epimedium* group displayed a better recovery of nerve function than that in the icariin and control groups, with better recovery in the icariin group than in the control group. The nerve pinch test showed that nerve regeneration was greater in the *Epimedium* group and the icariin group as compared to the control group. In addition, the muscle wet weight in the *Epimedium* group was significantly improved when compared with the icariin group, and the improvement in the icariin group was better than that in the control group at 8 weeks after operation. Our findings suggest that *Epimedium* extract effectively promotes peripheral nerve regeneration and improves the function of damaged nerves.

## 1. Introduction 

Treatment of peripheral nerve injury is a major challenge in clinical practice. With advances in molecular biology and development of microsurgical techniques and tissue engineering, peripheral nerve repair procedures have been greatly improved [1]. In the last 10 decades, most treatments for peripheral nerve injury in animal models have achieved histological and functional recovery. Approaches in humans, however, produce insufficient recovery, especially for proximal nerve injury [2–4]. The discrepancy in results from experiments and clinical trials mainly results from the longer distance between organ and points of damage in humans [5]. Moreover, the speed of nerve regeneration is relatively slow, and the regenerated axons often need 3 or sometimes up to 10 months to eventually grow into target organs and tissues. Therefore, long term treatment is essential when inducing nerve regeneration with neurotrophic factors [6]. Most neurotrophic factors are mainly the neuropoietic cytokines [7] such as nerve growth factor (NGF), brain-derived neurotrophic factor (BDNF), and neurotrophin (NF). However, only a few of these factors are used in clinical treatment because they often cause side effects, and the treatment with these factors is usually costly. Therefore, it is imperative to find other factors to promote peripheral nerve regeneration. Increasing attention has been paid to the traditional Chinese medicine (TCM) for promoting peripheral nerve regeneration [8–10] since these remedies often display effective clinical outcome, minor side effects, and effectiveness for multiple targets. Although TCM has complex ingredients and the specific pharmacological mechanisms for their effectiveness are still unclear, an effective clinical outcome is welcomed by many clinicians.

In our previous studies, results showed that systemic administration of a traditional formula, which mainly contains the Radix Hedysari, *Epimedium*, and so forth, could enhance the peripheral nerve regeneration in rats [11]. Thus, we hypothesized that *Epimedium* may be a major component promoting the peripheral nerve regeneration in the formula. *Epimedium* has been used in China to treat erectile dysfunction, postmenopausal syndrome, and osteoporosis for thousands of years [12]. Icariin is a major component of *Epimedium* [13, 14]. Based on findings in clinical pharmacodynamic studies on *Epimedium*, Shindel et al. have demonstrated that icariin can promote bone formation and has neurotrophic effects in both *in vivo* and *in vitro* experiments [15]. Tohda and Nagata also found that an extract of *Epimedium koreanum* could promote recovery of muscle function after spinal cord injury in rats [16]. In the present study, the effects of *Epimedium* extract and its main ingredient, icariin, on peripheral nerve regeneration were investigated.

## 2. Materials and Methods

### 2.1. Animals and Animal Model

A total of 42 healthy adult male SD rats weighing 200–220 g (SPF grade) were purchased from the Beijing Unilever Animal Co., Ltd., and housed in the Animal Center of People's Hospital of Peking University. Animals were maintained in a specific pathogen-free (SPF) environment with controlled humidity and 12:12 h light-dark cycle and were given *ad libitum *access to water and food. All efforts were made to minimize animal suffering and to reduce the number of animals used. All of the surgical procedures, experimental manipulations, and perioperative care were performed in strict accordance with the Chinese Guidelines for the Care and Use of Laboratory Animals and were approved by the Hospital Medical Ethics of Peking Union Medical Collage.

All the animals were randomly divided into 4 groups: sham group (*n* = 6), control group (*n* = 12), *Epimedium* extract group (*n* = 12), and icariin group (*n* = 12). Rats were anesthetized with a single intraperitoneal injection of 2% pentobarbital solution (30 mg/kg). In the control, *Epimedium* extract, and icariin groups, the right sciatic nerve was exposed, clamped at 5 mm above the first branch of the nerve for 1 min, and marked with a 10-0 nylon microscopic suture in the epineurium under aseptic conditions. In the sham group, the right tibial nerve was only exposed, without nerve crush. Animals in the sham and the control group were intragastrically treated daily with 1 mL of distilled water. Rats in the *Epimedium* extract group and the icariin group were intragastrically fed daily with 1 mL of *Epimedium* extract and icariin, respectively.

### 2.2. Preparation of *Epimedium* Extract and Icariin

*Epimedium* was purchased from Beijing Tong Ren Tang Pharmacy and icariin from China Pharmaceutical and Biological Products. Dried *Epimedium* leaves (1000 g) were immersed in pure water at a volume ratio of 1 : 10 and boiled for 1 h. This was repeated once and the supernatant was collected by using a 200-mesh gauze filter. The collected supernatant was kept at room temperature overnight to remove sediments. It was then concentrated to 1000 mL by boiling and kept at 4°C for use. Five grams of icariin was dissolved in 1000 mL of pure water and kept at 4°C for use.

### 2.3. Rat Sciatic Functional Index (SFI)

The sciatic functional index (SFI) was measured at weeks 1, 2, 4, and 8 after surgery. A walking track box (50 cm in length) was made and a white paper was cut to the appropriate dimensions and placed on the bottom. The hindlimbs were dipped into carbon ink and each rat was permitted to walk down the box in order to record the bilateral footprints 4-5 times. The following parameters were determined: (1) footprint length (PL), defined as the distance from the heel to toe; (2) toe spread (TS), defined as the distance from the first to fifth toes; and (3) intermediary toe spread (IT), defined as the distance between the second and fourth toes. The right footprints (E) were recorded and measured, and the left footprints served as controls (N). Three parameters were calculated: print length factor (PLF) = (EPL-NPL)/NPL, toe spread factor (TSF) = (ETS-NTS)/NTS, and intermediary toe spread factor (ITF) = (EIT-NIT)/NIT. These parameters were utilized to calculate the Bain-Mackinnon-Hunter (BMH) sciatic function index (SFI) as follows: SFI = −38.3 (PLF) ± 109.5 (TSF) ± 13.3 (ITF) − 8.8. The recovery rate of SFI was calculated as a score of 0–100. Zero represents normal and 100 as no function.

### 2.4. Nerve Pinch Test

Six rats were randomly selected from the control, *Epimedium* extract, and icariin groups for nerve pinch test at postoperative week 1. Rats were anesthetized with a single intraperitoneal injection of 2% pentobarbital solution (20 mg/kg). The right sciatic nerve was exposed again, and a clamp was gradually applied from the distal tibial nerve to the proximal tibial nerve. Then, the pain reflex was observed, and the distance between the farthest reflective site and the crushed site was recorded. Experiments in this study were double blind.

### 2.5. Immunofluorescence Staining of Growth Associated Protein-43 (GAP-43)

After nerve pinch test, animals were killed under anesthesia and sciatic nerves were collected from the 5 mm proximal crush point to the 20 mm distal point. Sciatic nerves were fixed in 4% paraformaldehyde for 6 h and then dehydrated in 15% sucrose for 8–12 h and 30% sucrose overnight. The nerves were embedded in OCT compound. At −20°C, 10 *μ*m sections were obtained. The slices were dried at room temperature for 2 h, kept at 4°C, fixed in acetone at −20°C for 20 min, and washed thrice with 0.3% triton-PBS (5 min for each). These sections were blocked with 10% normal goat serum for 1 h and incubated overnight with mouse anti-rat GAP-43 monoclonal antibody (1 : 500, Sigma) at 4°C. The slides were incubated with rabbit anti-mouse IgG-FITC antibody (1 : 100, Sigma) at room temperature for 1 h after being washed thrice with 0.3% triton-PBS (5 min for each). Sections were observed under a fluorescence microscope.

### 2.6. Electrophysiological Examination

At week 8, rats were anesthetized and sciatic nerves were exposed. Electrophysiological recordings were performed in a quiet room with an ambient temperature at 22-23°C. For the detection of compound muscle action potential (CMAP), the recording electrodes were located on the central portion of the triceps and reference electrodes on the ipsilateral thigh muscle. Paraffin was applied around the neural stem to reduce fluid pathway conduction. CMAP was recorded after a stimulus with the intensity of 0.9 mA was given, using a pulse width of 0.1 ms and a frequency of 1 Hz. The latency of CMAP was recorded after stimulation of the nerve at crushed site and 30 mm proximal from the sciatic nerve, and the nerve conduction velocity (NCV, m/s) was obtained semiautomatically by dividing the distance between two stimulating sites by the difference in the onset latency.

### 2.7. Measurement of Muscle Wet Weight and Histological Staining

Experimental rats were sacrificed by overdose anesthesia after electrophysiological test. Bilateral triceps were carefully separated, collected, and weighted as the muscle wet weight. The ratio of right weight to left weight was calculated. A 5 mm muscle was harvested with a scalpel blade from the middle of the muscles, formalin-fixed, and paraffin-embedded for HE staining. The morphology of muscles was observed under a microscope.

### 2.8. Statistical Analysis

Data were expressed as mean ± standard deviation. Comparisons of means among different groups were done using one-way analysis of variance (ANOVA). A value of *P* less than 0.05 was considered statistically significant.

## 3. Results

### 3.1. SFI

**Figure 1:**
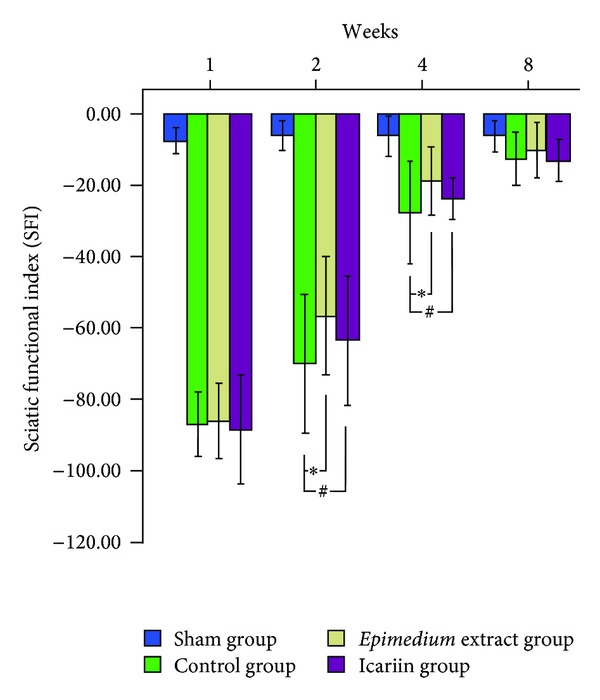
Effect of *Epimedium* extract and icariin on rat sciatic functional index (SFI). At week 2 and week 4, the SFI values are significantly better in rats of the *Epimedium* group than those of the control group. Moreover, the icariin group is better than the control group. At week 8, the sciatic functional index was not significantly different among the control, *Epimedium* extract and icariin groups. *P < 0.05, the *Epimedium* group versus the control group; ^#^ *P* < 0.05, the icariin group versus the control group (*n* = 6).

The SFI of the sham, control, *Epimedium* extract, and icariin groups was −7.54 ± 1.75, −86.95 ± 4.54, −85.98 ± 5.30, and −88.39 ± 7.64, respectively, at week 1; −5.88 ± 2.03, −70.08 ± 9.71, −56.65 ± 8.36, and −63.58 ± 9.23, respectively, at week 2; −6.32 ± 2.90 −27.50 ± 7.22, −18.61 ± 4.91, and −23.79 ± 3.03, respectively, at week 4; −6.15 ± 2.24, −12.71 ± 3.79, −10.20 ± 3.98, and −13.12 ± 2.94, respectively, at week 8. In the control, *Epimedium* extract, and icariin groups, SFI showed a gradual recovery of sciatic function, but the sciatic function was worse in the control group. At weeks 2 and 4, the neurological function was significantly improved in the *Epimedium* group and the icariin group as compared to the control group. At week 8, the SFI was comparable among these groups (Figure 1).

### 3.2. Nerve Pinch Test

**Figure 2:**
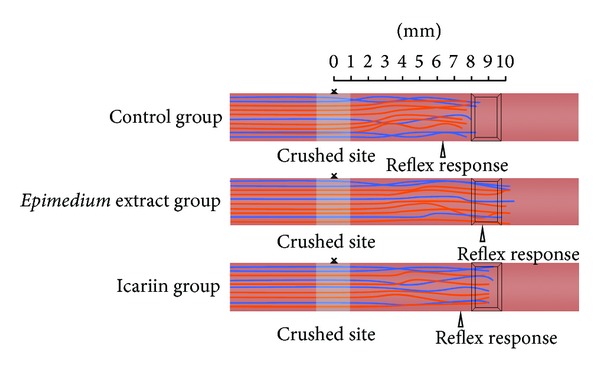
Nerve pinch test was performed to evaluate *Epimedium* extract and icariin effect on nerve regeneration. The arrow indicates the nerve reflective site. The average distances from the crushed site to the reflective site were 6.02 ± 0.64 mm, 8.07 ± 0.71 mm, and 6.58 ± 1.03 mm in the control, *Epimedium*, and icariin groups at week 1 after the operation.

At week 1, nerve pinch test showed that the average distance from reflective site to crushed site in the control, *Epimedium* extract, and icariin groups was 6.02 ± 0.64 mm, 8.07 ± 0.71 mm, and 6.58 ± 1.03 mm, respectively. The longest distance was found in the *Epimedium* extract group. The distance in the *Epimedium* extract group was significantly longer than that in the control group (*P* = 0.002) and the icariin group (*P* = 0.026), but no significant difference was observed between the icariin group and the control group (*P* = 0.303) (Figure 2).

### 3.3. GAP-43 Immunofluorescence Staining

**Figure 3:**
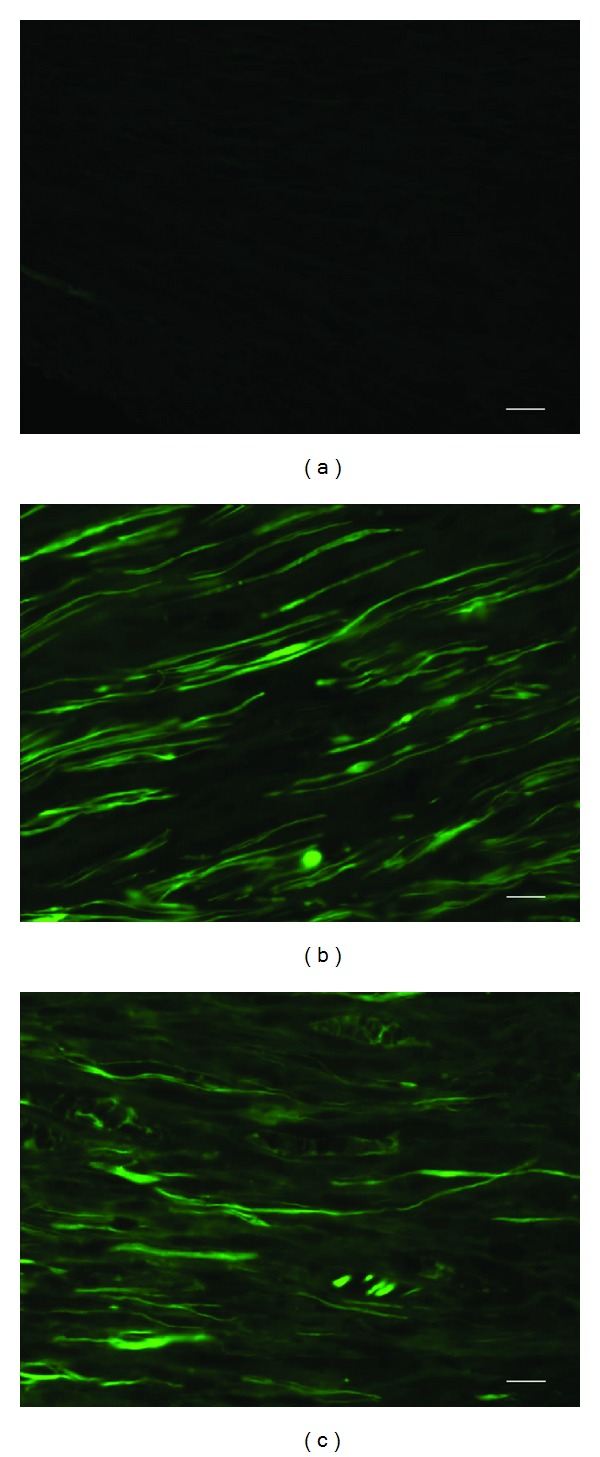
GAP-43 immunofluorescence staining was observed at 9 mm proximate to the crushed site in the control, *Epimedium*, and icariin groups at week 1 after the operation. (a) GAP-43 staining was hardly seen in the control group. (b) Evident GAP-43 staining was observed in the *Epimedium* extract group. (c) In the icariin group, GAP-43 staining was detectable but the density was lower than the *Epimedium* extract group. Scale bar: 5 *μ*m.

GAP-43 staining was performed to evaluate the regeneration of nerve fibers. GAP-43 expression was obviously observed at the reflective site. At the same site, 9 mm proximate to the crushed site, GAP-43 expression was significantly lower in the control group than in the *Epimedium* extract and icariin groups at week 1 after operation (Figures 3(a), 3(b), and 3(c)). It is indicated that the nerve fiber regeneration was better in the *Epimedium* extract group.

### 3.4. Nerve Electrophysiological Measurements

The nerve conduction velocity of the sham, control, *Epimedium* extract, and icariin groups was 47.25 ± 2.91 m/s, 28.22 ± 3.55 m/s, 32.02 ± 2.37 m/s, and 29.51 ± 3.29 m/s, respectively, at week 8 after operation. The nerve conduction velocity of the control group was significantly lower than that of the *Epimedium* extract group (*P* = 0.044). There was no significant difference between the *Epimedium* extract and the icariin groups (*P* = 0.17).

### 3.5. Muscle Wet Weight and Histological Staining

The bilateral wet muscle weight of the sham group was comparable. Atrophy of the muscle was evident in the control group, however, as compared to the *Epimedium* extract and the icariin groups. The wet weight of left normal muscle in the sham, control, *Epimedium* extract, and icariin groups was 1.285 ± 0.098 g, 1.271 ± 0.088 g, 1.303 ± 0.138 g, and 1.307 ± 0.086 g, respectively, showing no significant difference between any two groups. The wet weight of right muscle was 1.283 ± 0.071 g, 0.853 ± 0.042 g, 1.075 ± 0.111 g, and 1.010 ± 0.109 g in the sham, control, *Epimedium* extract, and icariin groups, respectively. The recovery rate of wet muscle weight was 100.10 ± 5.10%, 67.26 ± 3.67%, 82.56 ± 4.35%, and 77.13 ± 3.79% in the sham, control, *Epimedium* extract, and icariin groups, respectively. The muscle wet weight and recovery rate of the control, *Epimedium*, and icariin groups were lower than those of the sham group. The wet muscle weight and recovery of the control group were lower than those in the *Epimedium* group and the icariin group, and the muscle recovery rate in the *Epimedium* extract group was better than that in the icariin group.

**Figure 4:**
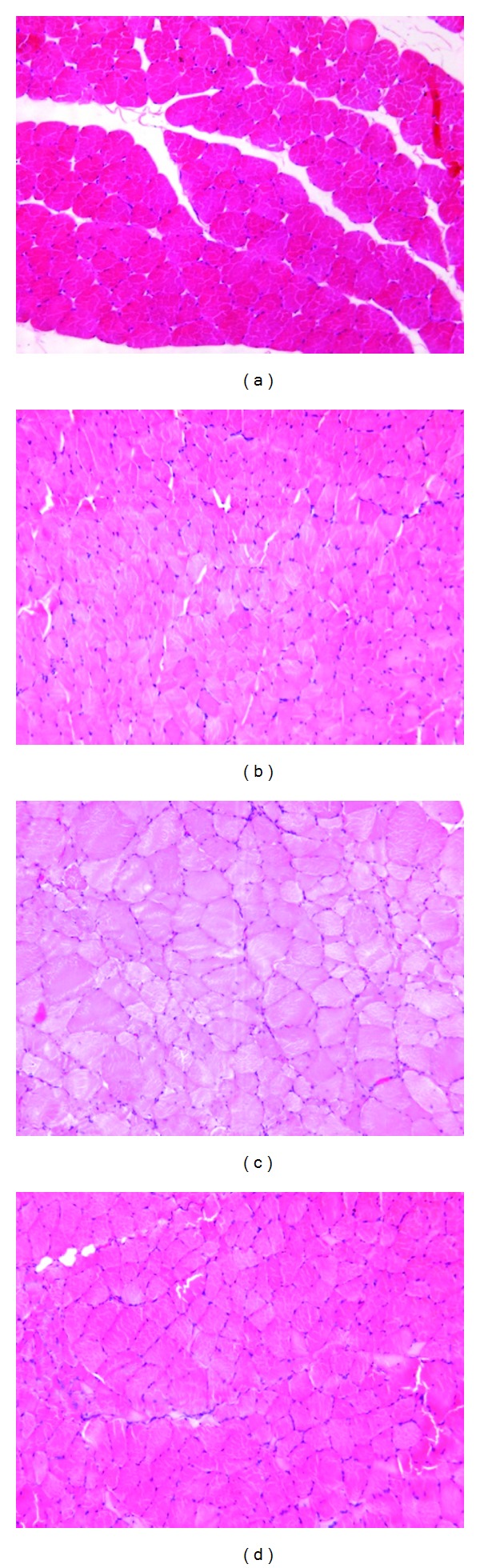
HE staining of muscle histology. (a) The sham group; (b) the control group; (c) the *Epimedium* extract group; (d) the icariin group.

The HE staining of normal muscle in the sham group showed clear boundaries of muscle fibers with uniform staining and similar diameter. In the control, *Epimedium* extract, and icariin groups, muscle fiber staining was not uniform, and the muscle diameter was smaller than that in sham group. Muscle diameter of the control group was the smallest (Figure 4).

## 4. Discussion

The SFI most commonly employed is the BMH SFI formula [17–19]. In this formula, an SFI of 0 is normal, and an SFI of −100 indicates complete impairment. In this study, the SFI was significantly improved in the *Epimedium* group and the icariin group as compared to the control group at 2 and 4 weeks after operation, suggesting that *Epimedium* extract and icariin are able to accelerate the functional recovery following injury. The extent of axonal regeneration distal to the injured site was also measured at week 1 by using the nerve pinch test and GAP-43 staining (staining of regenerating axons). Results demonstrated that *Epimedium* extract accelerated axonal growth, which is helpful to explain why the SFI in the *Epimedium* group was significantly improved at 2 and 4 weeks after operation as compared to the control group. Findings in nerve electrophysiological measurement, muscle wet weight measurement, and histological staining also supported that both *Epimedium* extract and icariin can exert protective effects on the motor function recovery and conductivity recovery in rats. It is presumed that icariin alone is effective to promote nerve regeneration, and may be one of the major components of *Epimedium* extract promoting the regeneration of peripheral nerves. However, the effects of *Epimedium* extract and icariin on the peripheral nerve regeneration are different, suggesting that components other than icariin in *Epimedium* extract may also facilitate the regeneration of peripheral nerves or coordinate this growth.

There are three animal nerve injury models frequently used in studies on the physiology and function of peripheral nerves: crush, transection, and graft/conduit [20, 21]. In this study, the sciatic nerve crush model, which has been employed in our previous studies, was used to investigate the influence of *Epimedium* extract on the peripheral nerve regeneration. This model is easy to establish and can be appropriately standardized, and it successfully reflects the restoration of nerve function. The sciatic nerve crush may produce a Sunderland type II injury, in which the myelin sheath and the axons are disrupted, but the basal lamina Schwann cell tubes remain intact [22]. Thus, the recovery of functional index is excellent and contrasts to the relatively poor recovery after nerve transection and repair. In this study, no difference in SFI was observed among these groups at 8 weeks after operation. This may be explained that the rat nerves achieve full restoration at 8 weeks after operation.

The dose of *Epimedium* extract used in this study was determined according to our previous studies [23]. The proportion of icariin in *Epimedium* extract was determined by high-performance liquid chromatography. The average proportion of icariin is about 4.873 mg/mL. Thus, the concentration of icariin prepared in this study was identical in both *Epimedium* extract and icariin solution.

Clinical and experimental studies have demonstrated that many TCM can promote peripheral nerve regeneration. For example, Wei et al. found that Hedysari extract (a TCM) prompted the regeneration of peripheral nerves in animal experiments [24]. Hsiang et al. also found that puerarin also promoted the regeneration of peripheral nerves [25]. In addition, Chen et al. showed that extract seemed to promote the PC12 differentiation and the regeneration of lateral nerve bud [26], efficiently improving the restoration of peripheral nerve function in experimental animals. Many molecular and pathophysiological changes are found to be involved in the process of nerve regeneration, which is in turn under the control of other factors. Recent studies have demonstrated that the interaction of multiple factors is important for the nerve regeneration and may exert better effects than single factor. For example, the nerve growth factors secreted by Schwann cells are a collection of multiple factors having more potent effects on the axonal growth and maturation of myelin than any individual factor. These studies suggest that the microenvironment involved in the nerve regeneration is really a collection of influences that can more efficiently promote the overall regeneration of peripheral nerves. Since TCM have multiple components in each treatment, they have the potential to effectively promote nerve regeneration. This was a preliminary study on the effect of *Epimedium* on nerve regeneration and growth, and the detailed mechanisms are still unclear. Further studies are warranted.
